# Multifocal visual evoked potential in thyroid associated ophthalmopathy

**DOI:** 10.1007/s10633-026-10113-x

**Published:** 2026-05-19

**Authors:** Mohamed Ibrahim Salah, Amany Abd Al-Fattah El-Shazly, Doaa Maamoun Ashour, Mahmoud Ahmed Elsamkary, Mohamed Nabil Hamza, Randa Hesham Ali Abdelgawad

**Affiliations:** Ophthalmology Department, Faculty of Medicine, Ain Shams University, Cairo, Egypt

**Keywords:** Thyroid associated ophthalmopathy, Multifocal visual evoked potential, Preclinical optic neuropathy, Electrophysiology in thyroid eye disease

## Abstract

**Purpose:**

To assess the visual function in patients with thyroid associated ophthalmopathy (TAO) without clinical optic neuropathy by multifocal visual evoked potential (mfVEP) and to correlate the findings with Humphrey visual field (HVF) and optical coherence tomography (OCT).

**Methods:**

This observational case–control study recruited a consecutive group of euthyroid TAO patients (excluding those with optic neuropathy) and a healthy matched control group. Ophthalmological assessment was done followed by HVF test, OCT retinal nerve fiber layer (RNFL) and ganglion cell complex (GCC), and mfVEP. For mfVEP, results were reported as mean signal-to-noise ratio (SNR) of upper and lower sectors of rings 5–6 (9.9°–22°).

**Results:**

Thirty patients were included in each group (TAO patients and controls). No significant differences were observed between the groups in RNFL thickness, GCC thickness, or upper/lower hemifield VF parameters. Analysis of the mfVEP revealed that, before multiple comparisons correction, SNR was lower in TAO patients. A repeated measures analysis of variance confirmed significant main effects for both patient group (*p* = 0.004), and test location (comparing SNR in the upper vs. lower sectors of rings 5–6) (*p* < 0.001), but no significant interaction.

**Conclusion:**

In this limited cohort, initial analysis of mfVEP results suggested reduction of the SNR in TAO patients without clinical optic neuropathy, but these findings did not remain statistically robust after adjustment for multiple comparisons. mfVEP alterations occurred despite normal OCT and visual field findings and warrant further evaluation in larger, longitudinal studies.

## Introduction

Optic neuropathy is a sight threatening complication affecting nearly 5% of patients with thyroid associated ophthalmopathy (TAO) [1]. This condition arises through several interacting components. Activation of orbital fibroblasts leads to fat deposition and fibrosis, resulting in the expansion of orbital tissues and proptosis [2]. This expansion causes crowding at the orbital apex, compressing the optic nerve and disrupting axonal transmission. In addition, evidence suggests that ischemic insult and inflammatory process contribute to the nerve dysfunction in TAO [3, 4]. Clinical diagnosis depends on the presence of relative afferent pupillary defect, optic nerve head edema, impairment of color vision, defective vision, and/or visual field (VF) defects. However, those signs are not easily detected in early disease or subclinical affection [2].

Various techniques were used aiming for early detection of structural and functional optic neuropathy in TAO before irreversible damage [5–7]. Early structural changes of the retinal nerve fiber layer (RNFL) in compressive optic neuropathy can be detected via ocular coherence tomography (OCT) [8]. While the automated VF has been widely accepted for detection and monitoring of functional optic nerve affection [6, 9]. The subjective results of the standard perimetric tests necessitates trial of different objective modalities for more reliable results. Accordingly, electrophysiological tests were introduced in the evaluation of TAO [10, 11].

Different electrophysiological tests were studied in TAO including flash and pattern visual evoked potential (VEP) as well as electroretinogram (ERG) [11]. Flash VEP can easily miss localized defects as it elicits a mass response from the anterior visual pathway. [12] Also, pattern VEP was found to be dominated in most individuals by responses from the lower field thus upper field defects can be missed [13]. Later, Baseler et al. introduced the concept of multifocal VEP (mfVEP) to provide objective topographic field mapping, however, they found limited clinical utility of this technique because of the great inter-subject variability of the obtained responses from identical locations in normal persons [14]. Later, Hood et al. suggested the use of interocular comparison to minimizes this variability [15]. Further improvements succeeded to make a clinical utility of a properly analyzed mono-ocular mfVEP [16–18]. Eliminating signals that were too noisy or too weak to measure posed another significant challenge. Several improvements were implemented until the introduction of the signal-to-noise ratio (SNR) as a measure of monocular amplitude, which enhanced the analysis of mfVEP recordings [19]. Hood et al. suggested that SNR must be taken in consideration for any comparison between Humphrey VF and mfVEP [17].

Owing to the previously mentioned improvements, mfVEP was used for objective visual field assessment in different optic nerve diseases including glaucomatous optic neuropathy and optic neuritis in multiple sclerosis [20, 21]. Accordingly, we designed this study to assess the visual function in patients with TAO without clinical manifestations of optic neuropathy by mfVEP and to correlate these mfVEP findings with the findings of automated perimetry and OCT.

## Methods

This is an observational case–control study that was conducted in Ain Shams University hospital. The study adhered to the tenets of the declaration of Helsinki, and the study protocol was approved by the research ethics committee of the Faculty of Medicine, Ain Shams University. All participants signed written informed consent after proper explanation of the study aim and procedure.

A consecutive sample of patients with TAO was recruited from the specialized oculoplastic clinic, Ain Shams University Hospital. Medical records were extracted and reviewed to ensure the diagnosis and to confirm the suitability for inclusion in this study. Inclusion criteria included diagnosis of TAO with previous laboratory evidence of hyperthyroid state and current euthyroid state. Patients with optic neuropathy were excluded. Other exclusion criteria included any systemic illness that could cause orbital and/ or optic neuropathy, refractive error more than ± 3, previous intraocular or orbital surgery, radiotherapy, media opacity, uveitis, or retinal disease. Recent deterioration of the best corrected visual acuity (BCVA) or color vision in the presence of relative afferent pupillary defect together with apical nerve compression in computerized tomography scan were diagnostic for optic neuropathy. Age and sex matched healthy volunteers were included as a control group. Only the right eye of each patient or control subject was included. A comprehensive medical and ophthalmological history was obtained, followed by ophthalmological assessment. This included BCVA measurement using the Snellen chart (with results converted to log MAR for analysis), pupil evaluation, slit-lamp examination, dilated fundus examination, and intraocular pressure (IOP) measurement. Ocular motility and proptosis assessments were also performed using Hertel’s exophthalmometer.

The following investigations were done for all participants:

### Humphrey visual field (HVF)

Visual field test was done using 24–2 Swedish interactive threshold algorithm (SITA) standard test by the Humphrey Field Analyzer II (Carl Zeiss Meditec, Inc., Dublin, CA). To account for the learning effect, the test was repeated within two weeks of the baseline examination. The second test was included in the analysis unless the patient had prior familiarity with the procedure. Only reliable results were accepted, defined as those with < 20% fixation losses and < 15% false-positive or false-negative rates. Testing was performed using a Goldmann size III stimulus with a background luminance of 31.5 apostilbs (asb). Recorded parameters included Mean Deviation (MD), and threshold sensitivity values (in dB).

### Optical coherence tomography (OCT)

Nidek RS-3000 Advance spectral domain OCT (Nidek, Gamagori, Japan; software version Navis-Ex 1.4.0.1) was used to the acquire the retinal nerve fiber layer thickness (RNFL). The machine default disc map protocol (6 × 6 mm) was used. Images were captured twice and images with better signal strength were analyzed. Subjects were excluded from the study if the OCT signal was less than 7/10. The whole average RNFL thickness in addition to averages of the upper and lower quadrants are automatically displayed. Also, average RNFL thickness in the four quadrants was displayed.

### Multifocal VEP

#### Recordings

The mfVEP was recorded monocularly for the right eye of each participant with RETI system (Roland Consult, Brandenburg, Germany). A standard pattern reversal dartboard stimulus was presented on a 19-inch TFT flat-screen. The stimulus was a 60 sector, each sector contained 16 checks, 8 black and 8 white, reversing at a regular frequency, using a pseudorandom m-sequence. Sectors and checks were scaled (scaling factor of 1.4) to be of approximately equal effectiveness, based upon cortical magnification factors.

Four active electrodes were placed on the subject’s skull using a cross-shaped electrode fixation device. One electrode was placed 3.5 cm above the inion, one 3 cm below, and two electrodes were placed 4 cm lateral to the inion on either side. The joint mounting of the electrodes ensured an equal distribution of their pressure and an improved electrode–skin contact. Thus, four differential recordings from electrode pairs with different orientations were derived. Biosignals were digitized with 1020 Hz sampling frequency and band-passed filtered was set between 1 and 30 Hz cut-off frequencies. The impedance level was set below 10 kΩ.

Subjects were corrected to the clearest vision at their viewing distance, taking into consideration their accommodative status. Accordingly, reading corrections were used when needed. With dilated pupils, the monocular response was recorded at 30 cm from the monitor. Subjects were instructed to fixate a cross at the center of the dartboard pattern (size 18°). Proper fixation was monitored with a video camera system. Every subject underwent four cycles (each lasting 141 s) to complete the acquisition protocol.

#### Response analysis

The signal data were processed with the system’s online analysis software. Further analysis was done off-line with MATLAB-based signal processing software (Matlab; Mathworks Inc., Natick, MA). To measure the mfVEP response amplitude, the root mean square (RMS) was calculated across the 0 to 500 ms interval. Subsequently, SNR was calculated for all stimulated fields and each of the four channels by dividing the RMS of the signal window between 50 and 150 ms by the average of the RMS values of the noise-only window between 230 and 330 ms. The software uses the best responses from the four channels, to generate a fifth channel plot response filtered using digital signal processor. The responses from this channel were then analyzed.

The primary outcome measure was the mean SNR for the upper and lower hemifields, calculated as the mean of the segments in rings 5 and 6 -located between 9.9° and 22°.

#### Correlation between results of HVF, OCT, and mfVEP

Humphrey Visual Field and mfVEP represent visual field data in fundamentally distinct formats, and no standardized method exists to integrate HVF decibel (dB) values across different retinal locations. To address this, we adopted a pragmatic approach as previously described by Afifi et al.[22]:

1. Visual Field Segmentation:
  Excluded the central four test points of the HVF (as these are not represented in mfVEP outputs).
  Divided the remaining field into upper and lower hemifields.
  Average values for upper and lower hemifields: All threshold values (x) expressed in dB were converted to their linear scale equivalents using the antilogarithmic transformation (10^(x/10)^). The resulting linear values were then averaged arithmetically. Finally, the mean linear value was converted back to the logarithmic scale to obtain the average value in dB for the upper and lower hemifields.
2. Quantitative Comparison:
  mfVEP: Calculated mean SNR for corresponding upper and lower hemifields were correlated to the averaged dB values calculated from the visual field upper and lower hemifields.
3. Anatomical Correlation:
  Upper hemifield results (HVF and mfVEP) were correlated with lower quadrant RNFL thickness measured by OCT, reflecting the anatomical projection of superior retinal axons to the inferior RNFL.
  Conversely, lower hemifield results were correlated with upper quadrant RNFL thickness.

### Statistical analysis

Data analysis was performed using the Statistical Package for the Social Sciences (SPSS) program, version 27. The normality of all continuous variables was assessed using the Shapiro–Wilk test. Data were expressed as Mean ± Standard Deviation (SD) for normally distributed variables and as Median (Interquartile Range [IQR]) for non-normally distributed variables. The independent sample Student t test was used to compare quantitative data between TAO patients and controls when both groups follow normal distribution while Mann–Whitney U tests were used for non-normally distributed data. Bonferroni-adjusted *p* values were calculated to adjust for multiple comparisons; significance set at 0.05. Spearmen’s correlation test was used to assess the association between pairs of quantitative variables. Repeated-measures analysis of variance (RM-ANOVA) test was used to evaluate SNR values across retinal locations (upper versus lower) and between study groups (TAO patients versus controls) as well as their interaction.

## Results

We included 30 patients in each group. The male/female ratio was 10/20 in the control group and 12/18 in the TAO patients’ group (χ2 = 0.29 and *p* = 0.592). There were no significant differences between the two study groups regarding age, SE, or C/D ratio (Table 1). However, BCVA was significantly lower in the TAO patients’ compared to the control group (Table 1).

**Table 1:** Clinical and demographic characteristics of the studied groups’ participants

	TAO patients		Controls		Test of significance*	*p* value	Adjusted* p* value**
	Median	IQR	Median	IQR			
Age (years)	43.00	39.00–60.75	43.00	38.25–62.50	z = 0.13	0.90	1.00
BCVA (LogMAR)	0.24	0.18–0.30	0.00	0.00–0.00	z = 6.22	< 0.001	< 0.001
SE (Diopters)	− 0.37	0.50 to − 0.25	− 0.25	− 0.50 to − 0.25	z = 1.34	0.182	1.00
IOP (mm Hg)	18.00	17.25–19.75	16.50	15.00–19.00	z = 2.14	0.033	0.49
C/D ratio	0.30	0.30–0.40	0.30	0.30–0.40	z = 0.91	0.369	1.00
Duration (years)	1.5	1.00–2.00			–		-
Degree of proptosis (mm)	22	20.00–23.00			–		-

*Mann–Whitney test of significance (z)

**p values were adjusted for multiple comparisons using the Bonferroni correction (15 comparisons)

BCVA: best corrected visual acuity, Log MAR: Logarithm of the Minimum Angle of Resolution, SE: spherical equivalent, IOP: intraocular pressure, C/D ratio: cup/disc ratio

Table 2 shows the OCT, VF, and mfVEP data for both groups. No significant differences in RNFL thickness, GCC thickness, and the VF parameters were found between the two groups.

**Table 2:** OCT RNFL, ganglion cell complex and field parameters in the thyroid associated ophthalmopathy (TAO) patients’ group versus the control group

	TAO patients		Controls		Test of significance	*p* value	Adjusted *p* value
	Median Mean	IQR ± SD	Median Mean	IQR ± SD			
Average RNFL (µm)	109.40	107.20–110.30	109.75	108.90–110.70	z = 1.74	0.084	1.00
Superior quadrant RNFL (µm)**	112.57	27.04	125.10	29.39	t = 1.49	0.091	1.00
Inferior quadrant RNFL (µm)**	119.53	15.02	128.90	23.99	t = 1.98	0.075	0.75
Superior ganglion cell complex (µm)	90.00	85.00–93.00	92.00	86.00–94.00	z = 1.40	0.165	1.00
Inferior ganglion cell complex (µm)	93.00	89.00–99.00	93.50	90.00–99.00	z = 0.07	0.947	1.00
MD (dB)	− 1.92	− 2.25 to − 1.46	− 1.72	− 2.23 to − 1.44	z = 0.85	0.399	1.00
VF upper (dB)	26.25	23.39–28.34	28.10	25.81–28.83	z = 1.70	0.141	1.00
VF lower (dB)	26.15	25.51–27.92	26.49	25.72–28.53	z = 0.91	0.328	1.00
Upper ring SNR	1.41	1.25–1.48	1.55	1.50–1.70	z = 2.31	0.007	0.39
Lower ring SNR**	1.64	0.24	1.79	0.26	t = 2.18	0.024	0.51

*Mann–Whitney test of significance (z)

**Student t-test of significance (t)

*p* values were adjusted for multiple comparisons using the Bonferroni correction (15 comparisons)

RNFL: retinal nerve fiber layer, MD: mean deviation, dB: Decibel, SNR: signal to noise ratio, VF: visual field

Primary analysis showed that the mean SNR of the upper and lower rings were statistically lower in TAO patients’ group than that of the controls, however, after correction for multiple comparisons both were statistically insignificant.

**Fig. 1:**
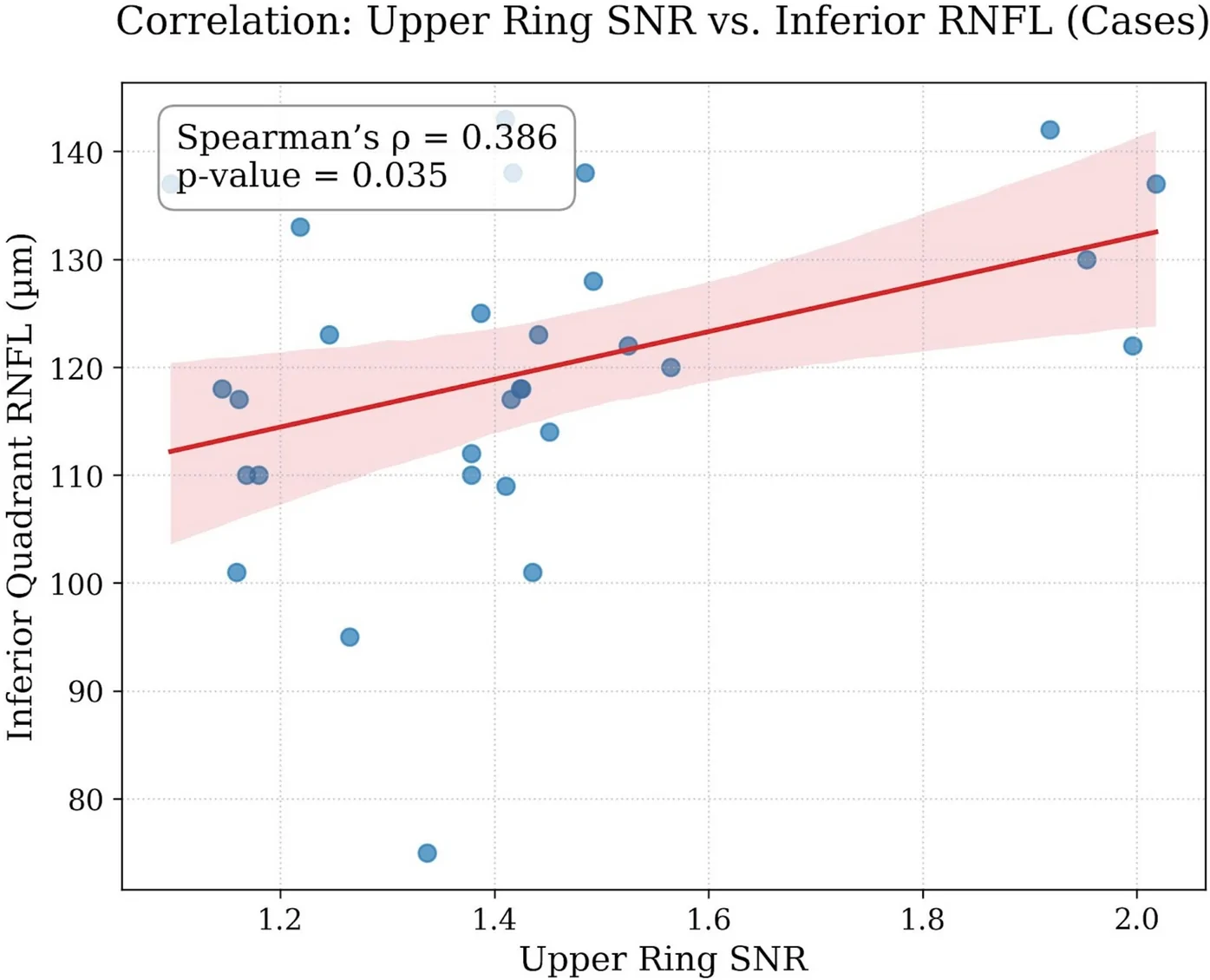
Correlation between upper ring signal-to-noise ratio (SNR) and inferior quadrant retinal nerve fiber layer (RNFL)

Table 3 shows correlations between the mean SNR of the upper and lower ring of the mfVEP and the corresponding visual field and OCT parameters. A weak to moderate positive significant correlation was found RNFL thickness in the inferior quadrant and the mean SNR of the upper ring (Fig. 1).

**Table 3:** Correlation analysis between mfVEP mean signal-to-noise ratio (SNR) of the upper and lower rings and corresponding OCT and VF parameters

	Spearman’s Correlation	*p* value	95% Confidence Intervals	
			Lower	Upper
Upper ring—Inferior quadrant RNFL (µm)	0.386	0.035	0.041	0.661
Upper ring—VF upper (dB)	0.107	0.572	− 0.275	0.443
Lower ring—Superior quadrant RNFL (µm)	− 0.066	0.727	− 0.469	0.335
Lower ring—VF lower (dB)	− 0.142	0.453	− 0.497	0.246

BCVA: best corrected visual acuity, Log MAR: Logarithm of the Minimum Angle of Resolution, SE: spherical equivalent, IOP: intra ocular pressure, RNFL: retinal nerve fiber layer, VF: visual field

A RM-ANOVA test was conducted with location (upper versus lower) as the within-subjects factor and group (TAO patients versus controls) as the between groups factor (Table 4). We found a significant main effect of location (F = 28.45, *p* < 0.001) as well as a significant effect of groups (F = 9.05, *p* = 0.004). While there was no significant interaction between location and groups.

**Table 4:** Repeated measures analysis of variance for signal-to-noise ratio (SNR) in upper and lower retinal rings in thyroid associated ophthalmopathy (TAO) patients and controls:

	TAO patients		Controls	
	Mean	SD	Mean	SD
Upper ring SNR	1.43	0.25	1.56	0.18
Lower ring SNR	1.64	0.24	1.79	0.26
F (location)	28.45			
*p* value (location)	< 0.001			
F (group)	9.05			
*p* value (group)	0.004			
F (interaction between group and location)	0.01			
*p* value (interaction between group and location)	0.89			

A repeated-measures analysis of variance was performed with retinal location (upper vs. lower) as the within-subjects factor and group (patients vs. control) as the between-subjects factor

## Discussion

Thyroid associated ophthalmopathy (TAO) can impair the optic nerve functions through various mechanisms that often lead to functional impairment before the appearance of clinically evident dysthyroid optic neuropathy. The present study evaluated the ability of mfVEP to detect early functional abnormalities in TAO patients without clinically evident optic neuropathy, and examined its relationship with HVF testing and OCT.

In our cohort of TAO patients, we observed a significant reduction in BCVA compared to healthy controls, occurring in the absence of clinical signs of overt optic neuropathy. This reduction is likely multifactorial. Ocular surface instability and corneal irregularities, secondary to proptosis (Median: 22 mm), may substantially degrade visual acuity. Previous studies have demonstrated that the instillation of artificial tears prior to visual acuity testing can improve results by stabilizing the tear film [23]; however, in the present study, BCVA was tested without prior lubrication. We recommend that future studies incorporate standardized surface lubrication to isolate neural from refractive deficits. Additionally, the reduced BCVA may reflect subtle, subclinical optic nerve dysfunction, as supported by the early changes detected in our mfVEP analysis.

In this cohort, no significant differences were observed between TAO patients and healthy controls with respect to RNFL thickness, GCC measurements, or standard VF parameters. These findings differ from the data published in TAO cases with clinically evident optic neuropathy. In a meta-analysis by Yang et al., the MD was significantly reduced in TAO patients with clinical optic neuropathy, and GCC was also significantly reduced, whereas, RNFL thickness showed no differences either in globally or across individual quadrants [24]. The median of the MD was − 1.92 dB for the patient group and − 1.72 dB for the control group. Although these values are slightly below the theoretical average of 0.00 dB, they remain within clinically normal limits. It is important to note that the lower baseline sensitivity in the control group -likely due to their relative lack of test experience (second session) compared to the more 'test-wise' patient group- could potentially mask early or subtle depression in the patient group. This finding aligns with research by Castro et al., who reported a nearly identical mean MD of − 1.73 dB in the second testing session of healthy individuals [25].

It is worthy of mention that the reduced BCVA observed in the patient group -despite within-normal VF parameters- may be attributed to the distinct physiological mechanisms of each test. Standard automated perimetry assesses differential light sensitivity using relatively large threshold stimuli and is generally less affected by mild refractive blur or ocular surface irregularities. Experimental work has demonstrated that 1.00 D of defocus causes only a modest generalized depression of the threshold values by 1.4 dB [26]. Therefore, the stability of the mean deviation in this cohort likely reflects the resilience of the visual field to minor optical disruptions that are nonetheless sufficient to degrade high-contrast spatial resolution.

Mean mfVEP SNR values in both upper and lower retinal rings were numerically reduced in the TAO group compared with controls. These differences reached statistical significance in the primary analysis; however, these differences did not remain statistically significant after adjustment for multiple comparisons. This emphasizes the subtle nature of functional changes in subclinical TAO and underscores the need for cautious interpretation of isolated mfVEP parameters. Nevertheless, RM-ANOVA analysis revealed a significant main effect of group, indicating an overall reduction in mfVEP responses across retinal locations in TAO patients. A significant main effect of retinal location was also observed, with higher SNR values in the lower ring in both TAO patients and controls; the clinical relevance of this finding warrants further investigation. The lack of a significant interaction between retinal location and group suggests that early functional impairment may be diffuse rather than confined to a specific hemifield.

Although the mfVEP findings did not remain statistically significant after correction for multiple comparisons, the observed functional alterations—where mfVEP suggested functional abnormalities in the absence of VF or OCT changes—aligns with established neuro-anatomical principles. It was previously found that standard perimetry can only detect VF defects after approximately 25–30% of retinal ganglion cells are lost [27]. These findings align with Pérez-Rico et al., who reported that 35.4% of eyes with Graves’ orbitopathy demonstrated significant alterations in mfVEP amplitudes and latencies despite normal structural and functional assessments [28]. Our findings are consistent with previous work, such as the study by Laron et al. in demyelinating optic neuritis, which also demonstrated the superior sensitivity of mfVEP over both VF and OCT, reinforcing its potential to identify the earliest stages of functional impairment [29]. Similarly, Xue et.al. studied the changes of mfVEP in cases of compressive optic neuropathy due to orbital tumors. They concluded that both mfVEP amplitude and latency were affected in a consistent manner with the visual field, except in few patients. mfVEP affection may appear earlier than the defect of visual field but the recovery is later than that of the field defect [27].

Correlation analyses provided additional insight into the potential relevance of mfVEP findings. Upper ring SNR showed modest but significant associations with inferior quadrant RNFL thickness, suggesting a relationship between electrophysiological function and subtle variations in optic nerve structure. However, the lack of consistent structure–function correlations across mfVEP parameters limits the interpretability of isolated findings and raises questions regarding the robustness of mfVEP as a standalone biomarker in subclinical TAO.

In contrast to our findings, which revealed no significant correlation between VF and mfVEP results, studies investigating optic neuropathies secondary to glaucoma or ischemia—such as those by Hood et al. [17] and Goldberg et al. [16] reported strong associations between these modalities. For instance, Hood et al. [19] demonstrated a linear relationship between mfVEP signal amplitude and the degree of visual field loss, a phenomenon likely attributable to progressive retinal ganglion cell damage.

In this context, it is noteworthy that the pattern visual evoked potential (PVEP) remains the most studied electrophysiological test in TAO. PVEP abnormalities, particularly prolonged P100 latency, correlate with reduced visual acuity, higher clinical activity score, and visual field defects [30, 31]. However, mfVEP offers the advantage of spatially localized functional assessment, which may enhance sensitivity for detecting early focal dysfunction. Nevertheless, mfVEP should be considered complementary to, rather than a replacement for, established electrophysiological and imaging techniques [12].

The mfVEP waveform consists of two principal components; C1: with a latency of ~ 65 ms, temporally analogous to the N75 component of PVEPs and C2: with a latency of ~ 95 ms, corresponding to the P100 component of PVEPs. This temporal alignment suggests shared neural generators between mfVEP and PVEP, though mfVEP’s spatial resolution enables finer localization of deficits [32]. Despite these similarities, Fortune and Hood concluded that the local mfVEP response is not simply a “little conventional VEP”. Specifically, that the C2 exhibits smaller amplitude and shorter latency (~ 95 ms for C2 vs. ~ 100–120 ms for P100). This divergence arises from methodological differences: the rapid stimulation sequence used in mfVEP likely diminishes contributions from extra striate cortical areas—which are more prominently engaged by slower, full-field PVEP protocols. Consequently, mfVEP responses might reflect earlier, more localized striate cortex activity, enhancing its sensitivity to focal dysfunction [13].

To our knowledge, only one prior study has evaluated mfVEP alterations in TAO without optic neuropathy. Perez-Rico et al. first investigated the application of mfVEP in TAO patients lacking optic neuropathy [28]. Our study replicated the parameters examined by Perez-Rico et al., while additionally incorporating an analysis of the ganglion cell complex (GCC).

In this preliminary study with a limited cohort, our findings suggest that mfVEP may reveal subtle functional alterations in TAO patients before the development of detectable VF or structural OCT changes. While these findings support a potential role for mfVEP as an adjunctive tool in the evaluation of early optic nerve involvement in TAO, they should be interpreted with caution given that statistical significance was attenuated after correction for multiple comparisons. A key advantage of mfVEP is its objective, quantifiable nature, which is particularly beneficial for TAO patients who may struggle with conventional perimetry due to severe proptosis, diplopia, or other physical limitations; however, significant technical and methodological challenges must be addressed before it can be integrated into routine clinical practice.

Several limitations warrant consideration. The relatively small sample size limits statistical power and may reduce the ability to detect subtle between-group differences. Lack of follow-up precludes assessment of the prognostic significance of mfVEP abnormalities. In addition, mfVEP acquisition and analysis remain technically demanding, with inherent signal variability and a lack of standardized protocols and normative databases, which currently limit routine clinical implementation. Accordingly, larger, longitudinal studies incorporating multimodal functional and structural assessments are needed to better define the clinical utility and predictive value of mfVEP in TAO.

## Data Availability

Access to the data supporting the results in this article requires direct correspondence with the corresponding author.
